# The PRP6-like splicing factor STA1 is involved in RNA-directed DNA methylation by facilitating the production of Pol V-dependent scaffold RNAs

**DOI:** 10.1093/nar/gkt639

**Published:** 2013-07-22

**Authors:** Kun Dou, Chao-Feng Huang, Ze-Yang Ma, Cui-Jun Zhang, Jin-Xing Zhou, Huan-Wei Huang, Tao Cai, Kai Tang, Jian-Kang Zhu, Xin-Jian He

**Affiliations:** ^1^National Institute of Biological Sciences, Beijing 102206, China, ^2^College of Life Science, Beijing Normal University, Beijing 100875, China, ^3^Shanghai Center for Plant Stress Biology and Institute of Plant Physiology and Ecology, Shanghai Institutes for Biological Sciences, Chinese Academy of Sciences, Shanghai 200032, China and ^4^Department of Horticulture and Landscape Architecture, Purdue University, West Lafayette, IN 47907, USA

## Abstract

DNA methylation is a conserved epigenetic marker in plants and animals. In *Arabidopsis*, DNA methylation can be established through an RNA-directed DNA methylation (RdDM) pathway. By screening for suppressors of *ros1*, we identified STA1, a PRP6-like splicing factor, as a new RdDM regulator. Whole-genome bisulfite sequencing suggested that STA1 and the RdDM pathway share a large number of common targets in the *Arabidopsis* genome. Small RNA deep sequencing demonstrated that STA1 is predominantly involved in the accumulation of the siRNAs that depend on both Pol IV and Pol V. Moreover, the *sta1* mutation partially reduces the levels of Pol V-dependent RNA transcripts. Immunolocalization assay indicated that STA1 signals are exclusively present in the Cajal body and overlap with AGO4 in most nuclei. STA1 signals are also partially overlap with NRPE1. Localization of STA1 to AGO4 and NRPE1 signals is probably related to the function of STA1 in the RdDM pathway. Based on these results, we propose that STA1 acts downstream of siRNA biogenesis and facilitates the production of Pol V-dependent RNA transcripts in the RdDM pathway.

## INTRODUCTION

Inactivation of transposons, retrotransposons and other DNA repeats is required for genome stability and integrity in plants as well as in fungi and animals (1–4). DNA methylation and repressive histone modification marks are critical for the silencing of these sequences (2,4). In *Arabidopsis*, DNA methylation mutations reactivate silenced transposable elements (TEs) and could lead to retrotransposition of the sequences, suggesting an important role of DNA methylation in maintaining genome stability and integrity (5–7).

DNA methylation in *Arabidopsis* is mainly established through an RNA-directed DNA methylation (RdDM) pathway (2,3). In the RdDM pathway, multi-subunit DNA-dependent RNA polymerases IV and V (Pol IV and Pol V) are responsible for generating 24-nt small interfering RNAs (siRNA) and nascent scaffold RNAs, respectively (8–14). NRPD1 is the largest subunit of Pol IV, whereas NRPE1 is the largest subunit of Pol V (12,14). Other subunits are shared or are unique in Pol II, Pol IV and Pol V (12,14). SHH1/DTF1 facilitates Pol IV occupancy on RdDM target loci and is required for the production of Pol IV-dependent 24-nt siRNAs (15,16). The RNA-dependent RNA polymerase RDR2 is proposed to be required for converting Pol IV-dependent RNA transcripts into double-stranded RNAs (17). Then, Dicer-like protein DCL3 cleaves the double-stranded RNAs into 24-nt siRNAs that are required for *de novo* DNA methylation (17). Twenty-four nucleotide siRNAs are loaded onto the ARGONAUTE protein AGO4 in the cytoplasm and then transported into the nuclei to form an RdDM effector complex, which recruits the *de novo* DNA methytransferase DRM2 to specific RdDM target loci on chromatin (18–20). Accumulation of Pol V-dependent scaffold RNAs relies on DRD1, DMS3, and RDM1, which form the DDR complex that facilitates Pol V function at RdDM target loci on chromatin (21–25). KTF1, a WG/GW motif-rich protein, can bind to nascent scaffold RNAs and associate with AGO4 (26,27). Simultaneous recruitment of KTF1 and AGO4 to specific RdDM genomic target loci is required for *de novo* DNA methylation (28).

In eukaryotes, the spliceosome machinery is responsible for the splicing of premature mRNAs (pre-mRNAs) by removal of introns and ligation of exons (29). The spliceosome is composed of five small nuclear ribonucleoprotein particles (snRNPs) U1, U2, U4, U5 and U6 (30). snRNPs are preliminarily assembled by the SMN (Survival of Motor Neuron) complex in the cytoplasm and transported to the nucleolus-adjacent Cajal body (31,32). The Cajal body is required for snRNP assembly and modification (31,32). After snRNPs exit the Cajal body, they are transported and assembled into spliceosome complexes on pre-mRNAs for splicing (30). U1 snRNP and U2 snRNP recognizes the 5′ splice site and the branch point sequence of the 3′ splice site, respectively. The subsequent addition of U4/U6-U5 tri-snRNP facilitates the formation of a mature spliceosome that catalyzes pre-mRNA splicing (33). Many non-snRNP-splicing factors are required for proper assembly of snRNPs and functional spliceosome complexes (34).

Consistent with the view that splicing-related proteins have multiple functions, splicing factors were demonstrated to be involved in several processes other than pre-mRNA splicing including transcription elongation, mRNA polyadenylation and telomerase RNA biogenesis and processing (35–37). In fission yeast, some splicing factors are involved in RNAi-induced transcriptional silencing (RITS) (38,39). The RITS pathway in fission yeast is similar to the RdDM pathway in *Arabidopsis*. The two processes share several evolutionarily conserved proteins including DNA-dependent RNA polymerases, RNA-dependent RNA polymerases, Dicers and Argonaute proteins (40,41). It is possible that the splicing factors in *Arabidopsis* are also involved in the RdDM pathway. In *Arabidopsis*, the SR family protein SR45 interacts with U1-70K as well as with the U2 snRNP auxiliary factor, suggesting that it has a role in spliceosome assembly (42,43). A recent report showed that SR45 is required for RdDM, although the detailed mechanism remains to be elucidated (44).

The *Arabidopsis* transgenic plants that harbor both *RD29A* promoter-driven luciferase transgene (*RD29A-LUC*) and *35S* promoter-driven kanamycin-resistance transgene *NPTII* (*35S-NPTII*) were previously generated. Both transgenes were expressed well in the wild-type background but were silenced when the active DNA demethylase gene *ROS1* was mutated (45). We identified several new regulators of transcriptional silencing by screening for suppressors of *ros1* based on the expression of either *RD29A-LUC* or *35S-NPTII* (46,47). In this research, we identified a PRP6-like splicing factor, STA1, by screening for suppressors of *ros1* and found that STA1 is required for RdDM and transcriptional gene silencing. We demonstrated that STA1 is required for accumulation of Pol V-dependent RNA transcripts. Moreover, STA1 colocalizes with AGO4 in the Cajal body and partially colocalizes with the largest subunit of Pol V, NRPE1 in the nucleoplasm. Based on these results, we propose that STA1 functions together with AGO4 and NRPE1 and has a critical role downstream of siRNA biogenesis in the RdDM pathway.

## MATERIALS AND METHODS

### Plant materials, map-based cloning and complementation testing

The *Arabidopsis* wild-type C24 and *ros1* mutant plants that contain both *RD29A-LUC* and *35S-NPTII* transgenes were used in this study. The expression of *RD29A-LUC* and *35S-NPTII* transgenes is silenced when the active DNA demethylase gene *ROS1* is mutated in *ros1*. The seeds of the *ros1* mutant harboring both transgenes were subjected to EMS treatment. We screened for suppressors of *ros1* in the EMS-mutagenized T2 library based on the expression of *RD29A-LUC* as determined by luminescence imaging. The identified mutant *ros1sta1* in the C24 background was crossed to the *ros1* T-DNA mutant in the Col-0 background. The F2 plants were used for map-based cloning. After the *sta1* mutation was mapped to a 220-kb interval, the *STA1* genomic DNA was sequenced, and a G to A mutation was found in the *STA1* open reading frame. The *STA1* genomic DNA in frame to *3xFlag* was cloned into the vector modified from pCAMBIA1305. The *STA1-3xFlag* construct was transformed into *ros1sta1* for complementation testing. The previously identified *ros1nrpd1* and *ros1npre1* mutants were used in the study as controls (46). An alternative *sta1* mutant allele that was characterized previously was used to confirm the function of STA1 (48).

### DNA methylation analysis by chop-PCR and bisulfite sequencing

Genomic DNA was extracted and purified from indicated genotypes with the Qiagen DNA extraction kit or by the CTAB method. For chop-PCR, genomic DNA was digested with the DNA methylation sensitive enzymes HaeIII and Alu, followed by amplification of *AtSN1* and *solo LTR*, respectively. The same sequences were amplified from corresponding undigested genomic DNA as controls. For bisulfite sequencing, 2 µg of genomic DNA was treated with the sodium bisulfite reagent (Qiagen, USA) to convert unmethylated cytosine into uracil. The converted DNA was purified and subjected to amplification with the primers indicated in Supplementary Table S1. The amplified DNA was cloned into the T-vector and sequenced. For each genotype, at least 15 clones were sequenced, and the percentages of methylated cytosines at CG, CHG and CHH sites were separately calculated. The DNA oligonucleotides used for chop-PCR are listed in Supplementary Table S1.

### Analyses of RNA transcripts and small RNAs

Total RNA was extracted with Trizol from 2-week-old seedlings or from flowers. The oligo-dT or sequence-specific primers were used for reverse transcription. Single-stranded cDNA was subjected to semiquantitative RT-PCR or quantitative RT-PCR. RNA transcript levels were determined by the PCR products. *TUB4* or *ACT7* was amplified as an internal control. Amplification of RNA samples was carried out to detect whether the RNA was contaminated with DNA. Small RNA was extracted and run on a 15% polyacrylamide gel as previously described (46). The small RNA was electrotransferred to Hybond-N+ membrane (Amersham), and the blot was subjected to small RNA hybridization. The probes were from either PCR products or DNA oligonucleotides. PCR products were labeled with α-^32^P-dCTP, whereas DNA oligonucleotides were labeled with γ-^32^P-ATP. Small RNA hybridization was conducted in PerfectHyb buffer (Sigma) overnight at 38°C. The DNA oligonucleotides are listed in Supplementary Table S1.

### Immunolocalization

Nuclei were extracted from young *Arabidopsis* leaves as described (49). For immunolocalization, nuclei were fixed in 4% formaldehyde and applied to slides as previously described (50). The nuclei were treated with the blocking solution (3% BSA in PBS) and then incubated with primary antibodies overnight at 4°C. Each primary antibody was properly diluted in the blocking solution. After slides were washed, secondary anti-mouse Tetramethyl Rhodamine Isothiocyanate-conjugated antibody (TRITC) (Invitrogen) and anti-rabbit Fluorescein Isothiocyanate-conjugated antibody (FITC) (Invitrogen) were added, and the slides were incubated at 37°C. Chromatin was counterstained with 4′-6-Diamidino-2- phenylindole (DAPI). Images were acquired by SPINNING DISK confocal microscopy and then analyzed with Volocity software.

### Whole-genome bisulfite sequencing

Genomic DNA was treated with sodium bisulfite and subjected to high-throughput sequencing. The DNA library was subjected to high-throughput single-end sequencing by HiSeq 2000 (Illumina). After the sequence data were preliminarily processed, the clean and high-quality sequences were mapped to C24 reference genome sequence using Bisulfite Sequence Mapping Program allowing two mismatches. The Tair10 genome sequence was modified according to single nucleotide polymorphism (SNP) identified between C24 and Col-0 to obtain C24 reference genome sequence. Matched reads were included for calculation of DNA methylation according to the method described previously (51,52). For each cytosine site, methylation was calculated only when the site had at least 5-fold coverage. The DNA methylation level was represented by the percentage of reads reporting C relative to the total number of reads reporting T and C at cytosine sites in indicated regions. Annotations of genes and TEs were downloaded from The Arabidopsis Information Resource. Our analyses included gene bodies, TE bodies and 1 kb upstream gene promoters with >10% DNA methylation in at least one of the samples being compared. DNA methylation difference was evaluated by Fisher exact test, and only those loci with significant DNA methylation difference (*P* < 0.01) were selected for analyses. To determine the DNA methylation level of Pol IV-dependent siRNA regions, the *Arabidopsis* genome was divided into nonoverlapping 500-bp windows, and the DNA methylation levels of 500-bp Pol IV-dependent siRNA regions were shown by Box plot.

### Small RNA deep sequencing

In all, 18–30-nt small RNA reads were mapped to C24 reference genome sequence. Mapped 24-nt small RNA reads in non-overlapping 500-bp windows across *Arabidopsis* genome were normalized to the numbers of reads per million (RPM). The siRNA accumulation level in each 500-bp region was represented by normalized RPM and compared between the wild-type and each mutant. The 500-bp regions that show at least 5-fold reduction of 24-nt siRNAs in *nrpd1* relative to the wild-type were defined as Pol IV-dependent siRNA regions. Based on previous reports, mutation of NRPE1 and the other downstream components can only partially reduce accumulation at a subset of Pol IV-dependent 24-nt siRNAs (53). Thus, the criteria for NRPE1- or STA1-dependent siRNA regions are decreased to 2-fold reduction of 24-nt siRNAs caused by *nrpe1* or *sta1*. Normalized RPM in non-overlapping 500-kb windows was plotted across *Arabidopsis* chromosomes to visualize the genome-wide Pol IV-dependent siRNA pattern in each genotype.

## RESULTS

### Identification and characterization of *ros1#493*

**Figure 1.**
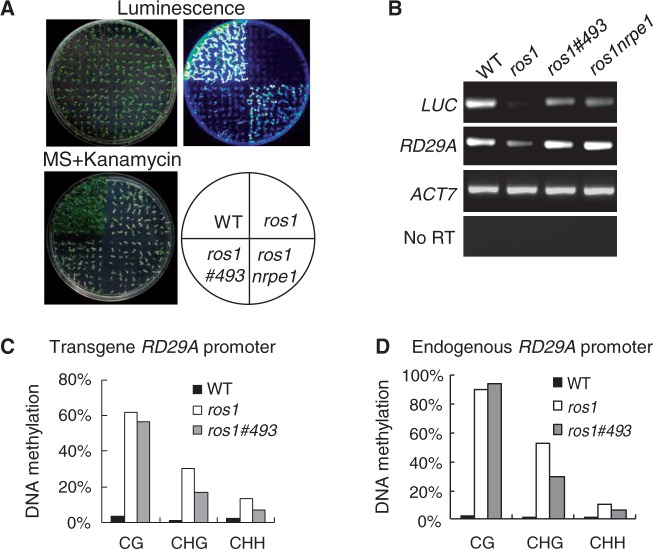
Effect of the *#493* mutation on DNA methylation and transcriptional silencing at the transgene and endogenous *RD29A* promoters. (**A**) The expression of the *RD29A-LUC* and *35S-NPTII* transgenes was evaluated by luminescence imaging and kanamycin resistance, respectively. Ten-day-old seedlings of indicated genotypes were incubated at 4°C for 5 days, followed by luminescence imaging. (**B**) Analyses of the *RD29A-LUC* transgene expression and the corresponding endogenous *RD29A* expression by semiquantitative RT-PCR. (**C** and **D**) Detection of DNA methylation at the transgene and endogenous *RD29A* promoters by bisulfite sequencing. The percentage of cytosine methylation at CG, CHH and CHH sites is shown for each genotype.

The previously characterized *RD29A* promoter-driven luciferase transgene (*RD29A-LUC*) and *35S* promoter-driven *NPTII* (*35S-NPTII*) are properly expressed in the wild-type background and are silenced in the *ros1* mutant (Figure 1A) (45). Many components required for transcriptional silencing were identified through screening for suppressors of *ros1* in T-DNA- or ethyl methanesulfonate (EMS)-mutagenized *ros1* libraries (46,47). The components in the RdDM pathway are specifically responsible for the silencing of *RD29A-LUC*, whereas the DNA replication-related proteins are only required for the silencing of *35S-NPTII* (47). From the EMS-mutagenized *ros1* library, we identified the *ros1#493* mutant in this study (Figure 1A). Luminescence imaging indicated that the silencing of *RD29A-LUC* was partially released in *ros1#493* and to a greater extent in *ros1nrpe1* (Figure 1A), which is consistent with the semiquantitative RT-PCR result (Figure 1B). Accordingly, the silencing of the endogenous *RD29A* gene was also released in *ros1#493* and *ros1nrpe1* (Figure 1B). The silencing of *35S-NPTII*, however, was not affected in *ros1#493* and *ros1nrpe1* (Figure 1A). The results suggest that, like the RdDM mutants, *#493* specifically releases the silencing of *RD29A-LUC* but not of *35S-NPTII*. To determine whether *#493* affects DNA methylation, we measured DNA methylation at the transgene and endogenous *RD29A* promoters in the wild-type, *ros1* and *ros1#493* by bisulfite sequencing. The results indicate that *sta1* reduces DNA methylation of both *RD29A* promoters at CHG and CHH sites (Figure 1C and D; Supplementary Table S2).

**Figure 2.**
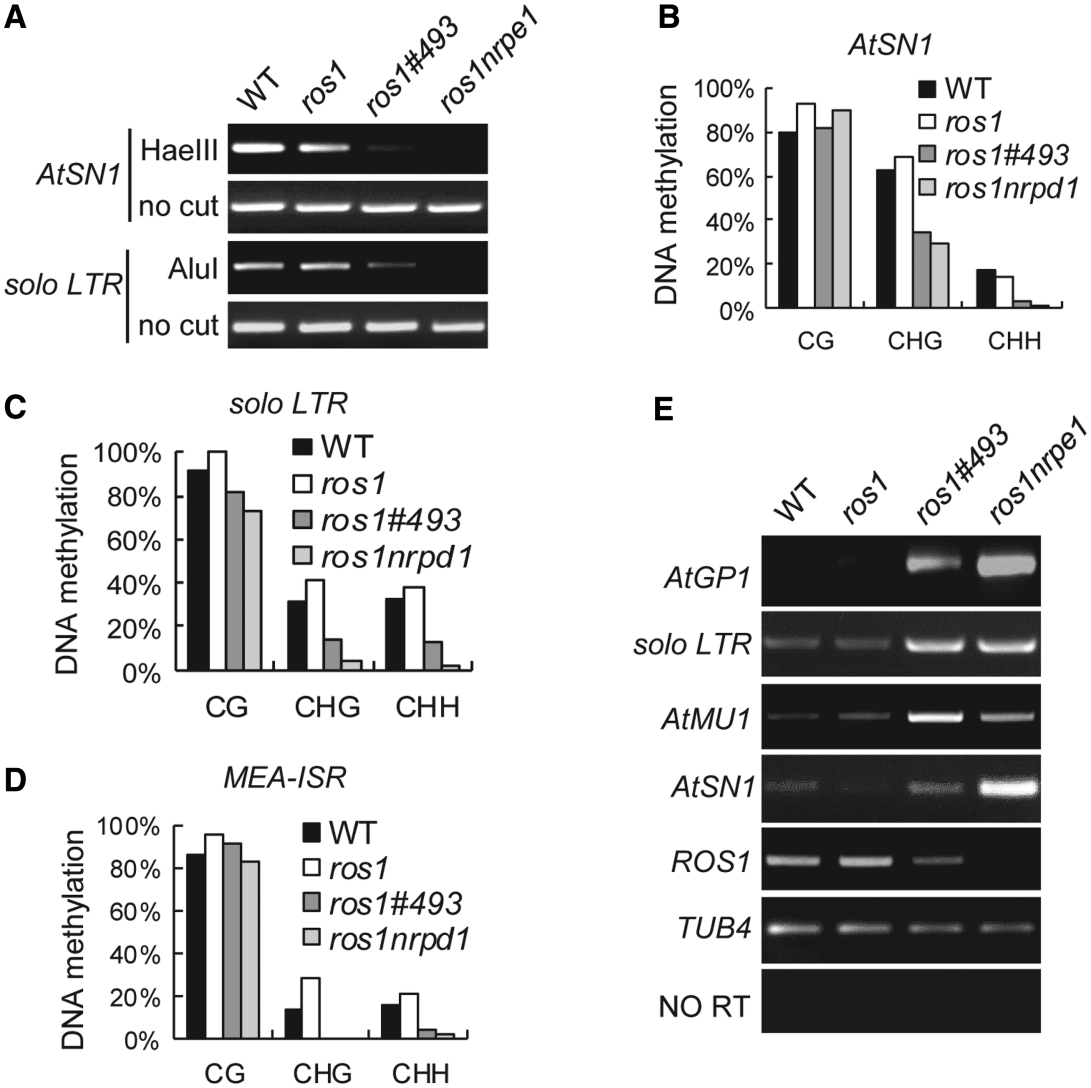
Effect of *sta1* on DNA methylation and transcriptional silencing at endogenous RdDM target loci. (**A**) DNA methylation analysis at *AtSN1* and *solo LTR* sites by chop-PCR. The genomic DNA from indicated genotypes was digested with HaeIII and AluI and subjected to amplification of *AtSN1* and *solo LTR*, respectively. (**B–D**) DNA methylation analysis at *AtSN1* (B), *solo LTR* (C) and *MEA-ISR* (D) by bisulfite sequencing. The percentage of methylated cytosine is shown for each type of cytosine context (CG, CHG and CHH). (**E**) The RNA transcripts of *AtGP1*, *solo LTR*, *AtMU1*, *AtSN1* and *ROS1* were measured by quantitative RT-PCR. *TUB4* was amplified as an internal control. Without reverse transcription (No RT), *TUB4* is unable to be amplified from RNA samples, suggesting the lack of DNA contamination.

To evaluate the effect of *#493* on DNA methylation at canonical endogenous RdDM target loci, we measured DNA methylation of *AtSN1* and *solo LTR* by chop-PCR. The genomic DNA from indicated genotypes was digested with the DNA methylation-sensitive restriction enzymes HaeIII and Alu I, and the digested DNA was used to amplify *AtSN1* and *solo LTR*, respectively. The results indicate that the DNA methylation of *AtSN1* and *solo LTR* is lower in *ros1#493* than in the wild-type and *ros1* and that the reduction of DNA methylation is less in *ros1#493* than in *ros1nrpe1* (Figure 2A). Bisulfite sequencing analysis shows that DNA methylation of *AtSN1* and *solo LTR* is reduced in *ros1sta1*, especially at CHG and CHH sites (Figure 2B and C; Supplementary Table S2). Moreover, according to bisulfite sequencing, the *MEA-ISR* methylation is markedly reduced at CHG and CHH sites in *ros1#493* as well as in *ros1nrpd1* (Figure 2D; Supplementary Table S2). These results suggest that the *#493* mutant and the RdDM mutants *nrpd1* and *nrpe1* have a similar effect on DNA methylation at canonical RdDM target loci.

Semiquantitative RT-PCR was performed to determine whether *sta1* affects the silencing of endogenous genomic target loci of RdDM. The results reveal that the silencing of *AtGP1*, *solo LTR* and *AtMU1* is substantially released in *ros1#493* as well as in *ros1nrpe1*, whereas the silencing of *AtSN1* is only mildly released in *ros1#493* relative to *ros1nrpe1* (Figure 2E). Because *#493* reduces the DNA methylation levels of RdDM target loci, the effect of *#493* on transcriptional silencing is correlated with reduced DNA methylation caused by *#493* (Figure 2E). Previous reports revealed that the *ROS1* transcript level is reduced by canonical RdDM mutations (54,55). Our result indicated that the *ROS1* transcript level is also reduced by *#493*, although the effect is less than that of *nrpe1* (Figure 2E), supporting the inference that *#493* affects transcriptional silencing through the RdDM pathway.

To identify the *#493* mutation, we crossed the *ros1#493* mutant in the C24 background with the *ros1* T-DNA mutant (Salk_045303) in the Col-0 background and used the F2 plants for map-based cloning. The *#493* mutation was localized to a 220-kb interval at the top end of Chromosome 4 (Supplementary Figure S1A). Sequencing of the *ros1#493* genomic DNA revealed a G to A mutation in the *STA1* opening reading frame (Figure S1B). STA1 is a conserved PRP6-like splicing factor that is required for pre-mRNA splicing as well as for mRNA stability (48). The STA1 protein contains three conserved domains: PRP6-N domain, HAT repeats and TPR domain (Supplementary Figure S1B). In the *ros1#493* mutant, the G to A mutation leads to the substitution of Gly413 with Asp in the HAT repeats domain of STA1 (Supplementary Figure S1B).

To confirm the function of STA1, we obtained an alternative *sta1* mutant allele that was identified previously (48). We found that the transcriptional silencing of the RdDM targets *AtGP1* and *solo LTR* is also suppressed in this *sta1* mutant allele (Supplementary Figure S2A and B), which is consistent with the notion that STA1 is involved in the RdDM pathway. We performed a complementation test to determine whether *STA1* is responsible for DNA methylation and transcriptional silencing. The full-length *STA1* genomic DNA in frame with *3xFlag* (*STA1-3xFlag*) was cloned into the vector modified from pCAMBIA1305. The *STA1-3xFlag* construct was transformed into *ros1sta1* for complementation testing. We found that the silencing of *RD29A-LUC* transgene is restored by the *STA1* transgene in *ros1#493* based on either luminescence imaging or semiquantitative RT-PCR (Supplementary Figure S3A and B). Moreover, the silencing of endogenous *RD29A* as well as of the endogenous RdDM target loci *solo LTR*, *AtGP1* and *AtMU1* is also restored (Supplementary Figure S3B). We carried out chop-PCR to test whether the DNA methylation defects are complemented by the *STA1* transgene. The results indicate that reduced DNA methylation of transgene and endogenous *RD29A* promoters, *AtSN1* and *solo LTR* in *ros1#493* are restored in the *STA1* transgenic plants (Supplementary Figure S3C). The *ros1#393* mutant plants are smaller in size and height compared with the wild-type and *ros1* (Supplementary Figure S3D). We found that the development defects of *ros1#493* are also complemented by the *STA1* transgene (Supplementary Figure S3D). Thus, STA1 is not only required for DNA methylation and transcriptional silencing but also for development.

### Genome-wide effect of *sta1* on DNA methylation

**Figure 3.**
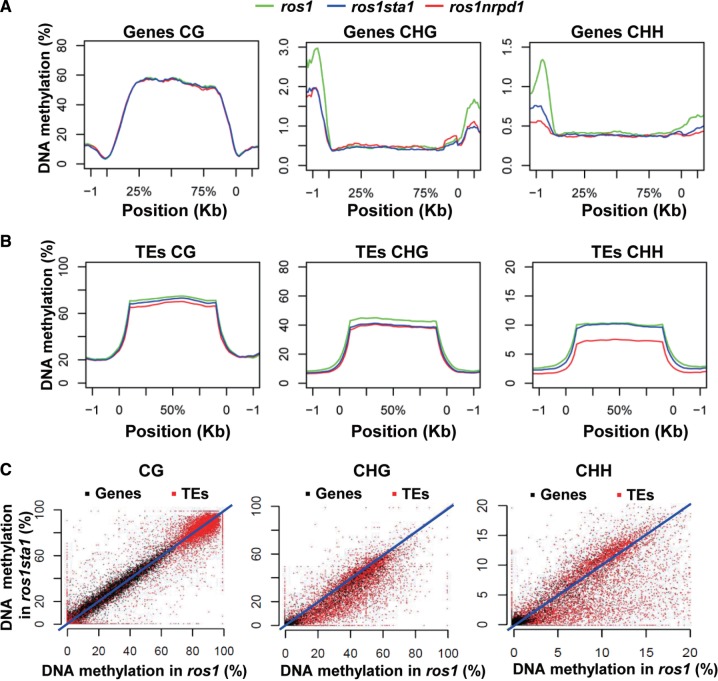
The genome-wide effect of *sta1* on DNA methylation of genes and TEs. (**A** and **B**) The levels of CG, CHG and CHH methylation across genes and TEs were plotted for *ros1*, *ros1sta1* and *ros1nrpd1*. The DNA methylation levels of genes and TEs as well as their 1-kb surrounding regions are shown. (**C**) The DNA methylation levels of genes and TEs in *ros1* and *ros1sta1*. The DNA methylation differences between *ros1* and *ros1sta1* are indicated by scattered dots.

We performed whole-genome bisulfite sequencing to investigate the genome-wide effect of *sta1* on DNA methylation and obtained 24 086 536, 21 664 677 and 24 009 978 reads from the libraries of *ros1*, *ros1nrpd1* and *ros1sta1*, respectively (Supplementary Table S3). Most of the reads from the three libraries are perfectly matched to the *Arabidopsis* genome, suggesting the high quality of the bisulfite sequencing results (Supplementary Table S3). The average DNA methylation patterns across genes and TEs were separately analyzed. Consistent with previous reports (56,57), we found that CG, but not CHG and CHH, is significantly methylated in gene bodies in each genotype (Figure 3A). The *sta1* and *nrpd1* mutations have no significant effect on the overall CG methylation level of gene bodies (Figure 3A). CHG and CHH methylation in gene surrounding regions is higher than that in gene bodies. Both *sta1* and *nrpd1* mutations reduce the overall CHG and CHH methylation levels in gene surrounding regions but have no effect on the overall gene body methylation level (Figure 3A). As expected, TE bodies are highly methylated at all three types of cytosine context compared with their surrounding regions (Figure 3B). Consistent with the preferential effect of the RdDM mutations on CHG and CHH methylation, CHG and CHH methylation of TEs is markedly reduced by *nrpd1*, whereas CG methylation is only marginally affected (Figure 3B). The *sta1* mutation causes a significant decrease in CHG methylation at TEs but has no clear effect on CG and CHH methylation on average at TEs (Figure 3B). However, a number of TEs that are hypomethylated at CHG and CHH sites were identified in *ros1sta1* versus *ros1* based on the whole-genome bisulfite sequencing data (Figure 3C), suggesting that *sta1* specifically affects CHG and CHH methylation at a subset of TEs (Figure 3C).

**Figure 4.**
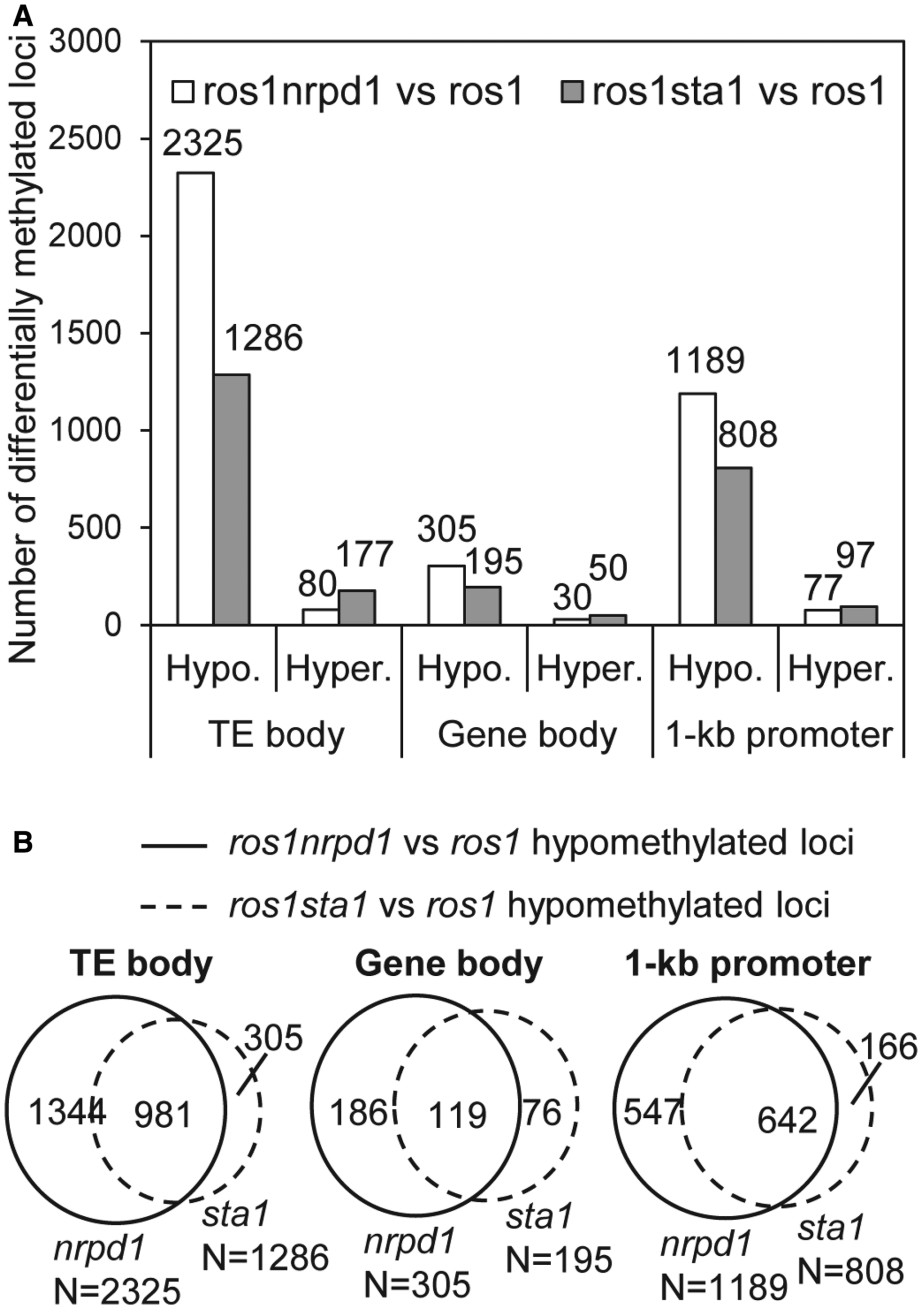
Differentially methylated loci in *ros1sta1* and *ros1nrpd1* relative to *ros1*. (**A**) The numbers of hypomethylated and hypermethylated loci were separately calculated based on whole-genome bisulfite sequencing data. The loci include TE bodies, gene bodies and 1-kb promoter sequences. (**B**) Venn diagrams show the numbers and overlaps of hypomethylated gene bodies, TE bodies and 1-kb promoters in *ros1sta1* and *ros1nrpd1* relative to *ros1*.

We calculated the numbers of differentially methylated gene bodies, TE bodies, 1-kb promoters (upstream of the transcript start) in *ros1sta1* and *ros1nrpd1* relative to *ros1* (Figure 4A; *P* < 0.01). We found that the number of hypomethylated TE bodies (2325) in *ros1nrpd1* versus *ros1* is much higher than the number of hypermethylated ones (80), which is consistent with the role of RdDM in TE methylation (Figure 4A; Supplementary Table S4). Like *nrpd1*, *sta1* also preferentially reduced TE methylation (Figure 4A). The number of hypomethylated TEs (1286) in *ros1sta1* versus *ros1* is significantly higher than that of hypermethylated ones (177) (Figure 4A; Supplementary Table S4). A high ratio of the hypomethylated TEs (989/1286) caused by *sta1* overlap with those caused by *nrpd1* (Figure 4B), suggesting that STA1 shares a number of common target TEs with the RdDM pathway. We found that 305 gene body regions are significantly hypomethylated in *ros1nrpd1* versus *ros1* (Figure 4A; Supplementary Table S5). This number is much less than the number of hypomethylated TEs (2325) caused by *nrpd1* in *ros1nrpd1*, supporting the inference that the RdDM pathway specifically functions on TE methylation. Compared with 305 hypomethylated gene bodies, only 30 gene bodies are significantly hypermethylated in *ros1nrpd1* versus *ros1* (Figure 4A; Supplementary Table S5), suggesting that *nrpd1* specifically reduces DNA methylation at a subset of gene bodies. The effect of *nrpd1* on DNA methylation of these gene bodies is likely related to the presence of TEs in either gene bodies or their flanking sequences. Like *nrpd1*, *sta1* reduces DNA methylation at 195 gene body regions in *ros1sta1* versus *ros1* (Figure 4A; Supplementary Table S5). Most of the hypomethylated gene bodies (119/195) caused by *sta1* overlap with the hypomethylated gene bodies caused by *nrpd1* (Figure 4B), confirming the function of STA1 in the RdDM pathway. Previous study suggested that the RdDM pathway is required for DNA methylation at the promoter sequences of a number of genes (25). Consistent with this study, we identified 1189 genes whose 1-kb promoter sequences are significantly hypomethylated in *ros1nrpd1* versus *ros1*, whereas 808 genes whose promoters are hypomethylated in *ros1sta1* versus *ros1* (Figure 4A; Supplementary Table S6). A high ratio of the hypomethylated promoters (642/808) caused by *sta1* overlap with those caused by *nrpd1* (Figure 4B). We found that the *SDC* promoter, a well-characterized RdDM target, is significantly hypomethylated in either *ros1nrpd1* or *ros1sta1* relative to *ros1* (Supplementary Table S6). These results demonstrate that STA1 as well as NRPD1 function on DNA methylation at the promoters of a subset of genes.

Our whole-genome bisufite sequencing results were confirmed by individual locus bisulfite sequencing analysis at three randomly selected hypomethylated loci (Supplementary Figure S4; Supplementary Table S2), demonstrating that our whole-genome bisulfite sequencing data are reliable. We investigated the effect of *sta1* on the global DNA methylation status across the five *Arabidopsis* chromosomes. The results suggest that the DNA methylation level is much higher at centromeric regions than at two arms of chromosomes in both *ros1* and *ros1sta1* (Supplementary Figure S5A–E). The *sta1* mutation preferentially reduces DNA methylation at pericentromeric regions but not at centromeric regions and two arms of chromosomes (Supplementary Figure S5A–E). Together, STA1 and the RdDM pathway share a large number of common targets in *Arabidopsis* genome. It suggests that STA1 contributes to DNA methylation through the RdDM pathway.

### Genome-wide effect of *sta1* on small RNA accumulation

**Figure 5.**
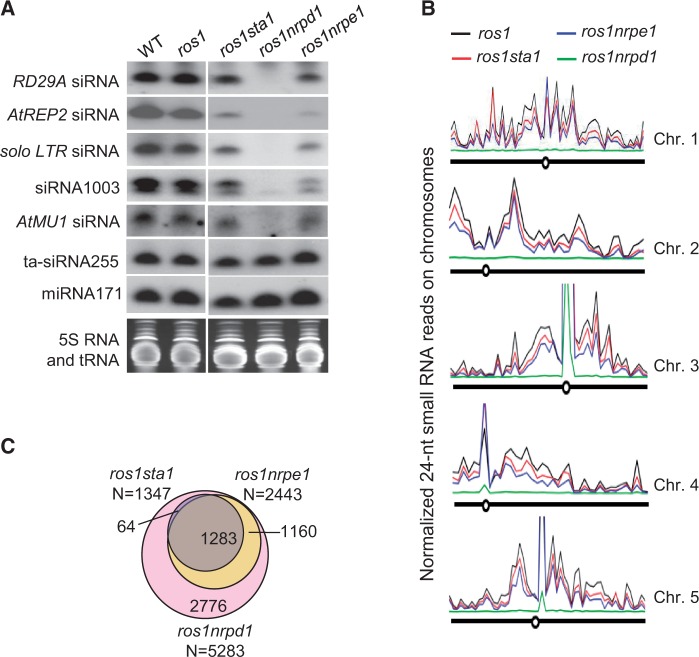
Effect of *sta1* on small RNA accumulation and RNA transcripts. (**A**) Effect of *sta1* on small RNA accumulation was detected by small RNA northern blotting. The accumulation of indicated 24-nt siRNAs was tested in the wild-type, *ros1*, *ros1sta1*, *ros1nrpd1* and *ros1nrpe1*. The ethidium bromide-stained small RNA gel was imaged as a loading control. (**B**) Effect of *sta1* and *nrpe1* on accumulation of Pol IV-dependent siRNAs on *Arabidopsis* chromosomes. Distribution of Pol IV-dependent siRNAs was plotted for *ros1*, *ros1nrpd1*, *ros1sta1* and *ros1nrpe1* based on normalized Pol IV-dependent 24-nt siRNA reads in non-overlapping 500-kb windows on each chromosome. (**C**) Shown are the numbers and overlaps of the 500-bp regions in which 24-nt siRNAs are reduced in *ros1nrpd1*, *ros1nrpe1* and *ros1sta1*.

We carried out small RNA northern blotting to investigate whether *sta1* affects accumulation of Pol IV-dependent 24-nt siRNAs. NRPD1, the largest subunit of Pol IV, is required for biogenesis of 24-nt siRNAs in the RdDM pathway (8,12). NRPE1, the largest subunit of Pol V, partially reduces accumulation of a subset of Pol IV-dependent siRNAs (53). Our results indicate that the Pol IV-dependent 24-nt siRNAs (*RD29A* siRNA, *AtREP2* siRNA, *solo LTR* siRNA, siRNA1003 and *AtMU1* siRNA) are all blocked in *ros1nrpd1*, whereas *RD29A* siRNA, *AtREP2* siRNA, *solo LTR* siRNA and siRNA1003, but not *AtMU1* siRNA, are partially decreased in *ros1nrpe1* (Figure 5A; Supplementary Figure S6), which is consistent with previously reports (23,26). The effect of *sta1* on Pol IV-dependent siRNA accumulation is comparable with that of *nrpe1* (Figure 5A; Supplementary Figure S6). Neither miRNA171 nor ta-siRNA255 is significantly affected by *sta1*, *nrpd1* and *nrpe1* (Figure 5A; Supplementary Figure S6).

In parallel with whole-genome bisulfite sequencing, we performed small RNA deep sequencing in *ros1*, *ros1sta1*, *ros1nrpd1* and *ros1nrpe1*. We calculated the siRNA levels of known RdDM target loci based on the small RNA deep-sequencing data. The result suggests that the siRNA levels determined by small RNA deep sequencing is highly consistent with those determined by small RNA northern blotting [Figure 5A; Supplementary Figures S6 and S7; (46)], suggesting that the small RNA deep-sequencing data are reliable. Pol IV-dependent siRNA levels across *Arabidopsis* chromosomes were plotted according to normalized small RNA reads in sliding 500-kb windows (Figure 5B). The results indicate that Pol IV-dependent 24-nt siRNAs are preferentially enriched at centromeric and pericentromeric regions across each *Arabidopsis* chromosome (Figure 5B), which is consistent with previous reports (53,58). Pol IV-dependent 24-nt siRNAs are markedly decreased in *ros1nrpd1* relative to *ros1*, whereas these siRNAs are partially decreased in *ros1nrpe1* and to a lesser extent in *ros1sta1* (Figure 5B). We identified 5283 siRNA regions in which 24-nt siRNAs are markedly decreased (>5-fold decrease) by *nrpd1* (Figure 5C; Supplementary Table S7). In these Pol IV-dependent siRNA regions, we identified 2443 Pol V-dependent siRNA regions (46.2%, 2443/5283; >2-fold decrease) and 1347 STA1-dependent siRNA regions (25.5%, 1347/5283; >2-fold decrease) (Figure 5C). In contrast, we found no Pol IV-dependent siRNA region where 24-nt siRNAs are increased by *sta1* and *nrpe1* (Supplementary Table S7), confirming the effect of *sta1* and *nrpe1* in 24-nt siRNA accumulation. We found that the accumulation of 24-nt siRNAs in 95.2% of the STA1-dependent siRNA regions depends on both NRPD1 and NRPE1 (Figure 5C; Supplementary Table S7), suggesting that *sta1* and *nrpe1* have a similar effect on Pol IV-dependent 24-nt siRNA accumulation. NRPE1, as the largest subunit of Pol V, is responsible for producing scaffold non-coding RNAs that are involved in the recruitment of RdDM effector complex at a later step of the RdDM pathway (9). The function of NRPE1 on 24-nt siRNA accumulation is likely caused by the feedback effect of NRPE1 on DNA methylation (53). It is possible that STA1 may also indirectly affect Pol IV-dependent 24-nt siRNA accumulation and act at a later step of the RdDM pathway.

### Effect of *sta1* on the DNA methylation at Pol IV-dependent siRNA regions and the production of Pol V-dependent scaffold RNAs

**Figure 6.**
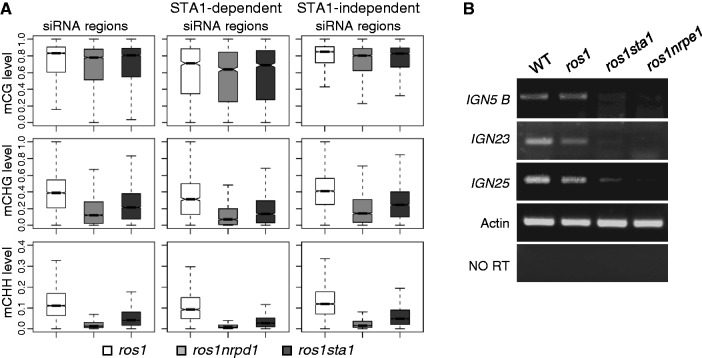
Effect of *sta1* on the DNA methylation of Pol IV-dependent siRNA regions and on the level of Pol V-dependent RNA transcripts. (**A**) DNA methylation levels of *ros1*, *ros1nrpd1* and *ros1sta1* at Pol IV-dependent siRNA regions were separately shown at CG, CHG and CHH sites by Box plot. Pol IV-dependent siRNA regions were divided into STA1-dependent siRNA regions and STA1-independent siRNA regions. DNA methylation levels at the two different regions are shown. (**B**) Effect of *sta1* on the level of Pol V-dependent RNA transcripts. Pol V-dependent RNA transcripts *IGN5 B*, *IGN23* and *IGN25* were measured by semiquantitative RT-PCR in WT, *ros1*, *ros1sta1* and *ros1nrpe1*.

We investigated whether the effect of *sta1* on Pol IV-dependent siRNA accumulation is related to DNA methylation changes. In the Pol IV-dependent siRNA regions, *sta1* as well as *nrpd1* only marginally affect CG methylation but markedly reduce CHG and CHH methylation in the Pol IV-dependent siRNA regions (Figure 6A), which again indicates that RdDM has a critical role in asymmetric CHH methylation. Moreover, the effect of *sta1* on CHG and CHH methylation is weaker than that of *nrpd1* (Figure 6A). To coordinate the genome-wide effects of *sta1* on siRNA accumulation and DNA methylation, we divided the Pol IV-dependent siRNA regions into two subgroups: STA1-independent siRNA regions and STA1-dependent siRNA regions. We found that *sta1* significantly reduces CHG and CHH methylation at STA1-dependent siRNA regions, supporting the notion that Pol IV-dependent siRNA accumulation is related to DNA methylation (Figure 6A). However, *sta1* as well as *nrpd1* can also reduce CHG and CHH methylation at STA1-independent siRNA regions (Figure 6A). Like previously described in *nrpe1* (53), the effect of *sta1* on Pol IV-dependent siRNA accumulation is secondary to DNA methylation.

Given that the function of STA1 on 24-nt siRNAs is similar to that of NRPE1 (Figure 5A–C; Supplementary Table S7), we hypothesized that, like NRPE1, STA1 may act together with NRPE1 and facilitate the production of Pol V-dependent scaffold non-coding RNAs. We conducted semiquantitative RT-PCR to test the effect of *sta1* on Pol V-dependent scaffold RNAs. *IGN5B*, *IGN23* and *IGN25* are three previously characterized Pol V-dependent scaffold RNAs (28). Our results indicated that the RNA transcripts from the three loci are substantially reduced in *nrpe1* (Figure 6B), which is consistent with the previous reports. The RNA transcripts are also reduced by *sta1*, although the reduction is less than that caused by *nrpe1* (Figure 6B). The results support the view that STA1 acts at a later step in the RdDM pathway and is required for producing Pol V-dependent scaffold RNAs.

### The relationship between STA1 and the canonical RdDM pathway

Because STA1 is a pre-mRNA splicing factor, involvement of STA1 in RdDM might be due to the splicing of genes encoding canonical components of the RdDM pathway. We performed RT-PCR to test potential splicing changes in major RdDM genes. If *sta1* causes a splicing defect in any RdDM genes, an unspliced transcript is expected to be detected by RT-PCR using intron-flanking primers (Supplementary Figure S8). The results indicate that no unspliced transcript is detected in *ros1sta1* relative to *ros1* (Supplementary Figure S9), suggesting that *sta1* is unlikely to indirectly affect RdDM through the splicing changes of RdDM genes. Moreover, our mRNA deep-sequencing data suggest that *sta1* does not decrease the transcript level of any major RdDM genes (Supplementary Table S8). The results, which were further confirmed by quantitative RT-PCR assay (Supplementary Figure S10), indicate that STA1 is likely directly involved in RdDM.

**Figure 7.**
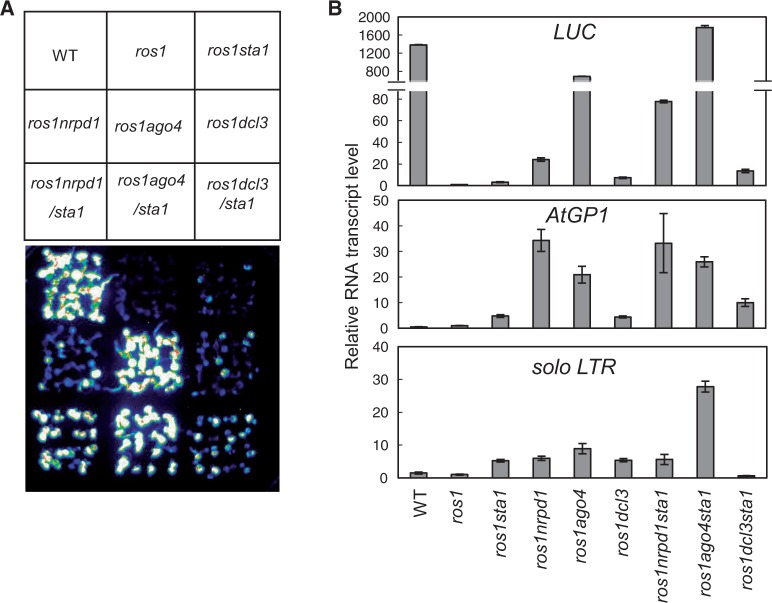
Analysis of the genetic relationship between *sta1* and known RdDM mutations. (**A**) The *sta1* mutation was separately introduced into *ros1nrpd1*, *ros1ago4* and *ros1dcl3* to determine the genetic relationship between *sta1* and different RdDM mutations. Luminescence imaging was conducted to detect the expression of *RD29A-LUC* in the indicated genotypes. (**B**) Quantitative RT-PCR was performed to measure the RNA transcript levels of *RD29A-LUC*, *AtGP1* and *solo LTR*.

Previous reports suggested that mutations of RdDM genes release the silencing of the *RD29A-LUC* transgene in the *ros1* background (23,26,46,59). We introduced the *sta1* mutation into *ros1nrpd1*, *ros1ago4* and *ros1dcl3* to evaluate the effect of *sta1* on transcriptional silencing in the triple mutants *ros1sta1nrpd1*, *ros1sta1ago4* and *ros1sta1dcl3*. Our luminescence assay indicated that the *sta1* mutation enhances the expression of *RD29A-LUC* in each of the three triple mutants, which is consistent with the quantitative RT-PCR results (Figure 7A and B). We further investigated the relationship between STA1 and the RdDM components at the endogenous RdDM target loci *AtGP1* and *solo LTR*. The results show that *sta1* has no effect on the transcript level of *AtGP1* in *ros1sta1nrpd1* or *ros1sta1ago4* but weakly enhances it in *ros1sta1dcl3* (Figure 7B). At *solo LTR*, *sta1* enhances the transcript level in *ros1sta1ago4* but not in *ros1sta1nrpd1* and *ros1sta1dcl3* (Figure 7B). The results suggest that the relationship between STA1 and the RdDM components varies depending on the RdDM target loci. When *sta1* shows no additive effect with RdDM mutants, STA1 may function directly through the corresponding RdDM components. When *sta1* has an additive effect with RdDM mutants, the function of STA1 at the RdDM target loci may be partially through an RdDM-independent pathway. Together, our results suggest that STA1 acts in transcriptional silencing possibly through both RdDM-dependent and -independent mechanisms.

### STA1 associates with splicing-related proteins and localizes in the Cajal body

We performed affinity purification of STA1-Flag from *STA1-Flag* transgenic plants. Copurified proteins were run on an SDS–PAGE gel and detected by mass spectrometry (Supplementary Figure S11). With two independent experiments, we identified a total of 15 copurified proteins that are related to pre-mRNA splicing (Supplementary Table S9); this supported the view that STA1 is a typical splicing factor. However, no known RdDM protein was identified from the affinity purification of STA1-Flag. The role of STA1 in the RdDM pathway needs to be further elucidated.

**Figure 8.**
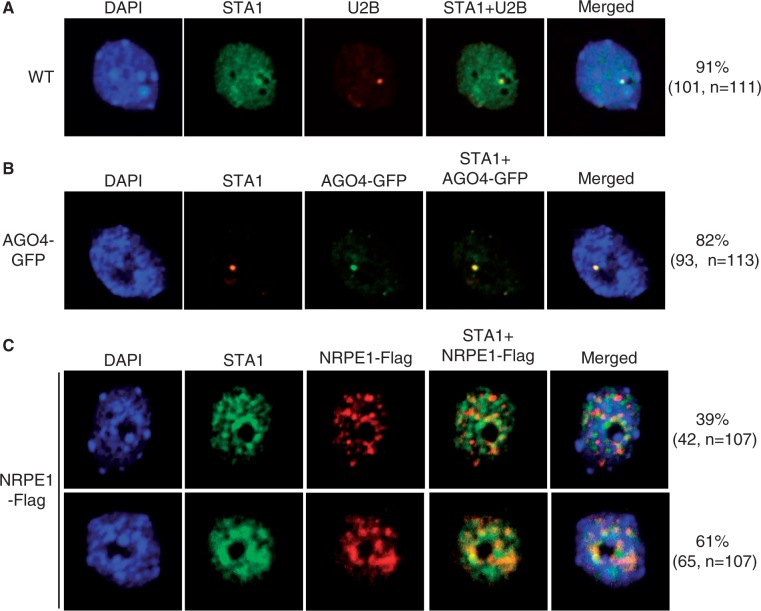
Immunolocalization of STA1 relative to the Cajal body, AGO4 and NRPE1 in nuclei. (**A**) Coimmunolocalization of STA1 and U2B in the nuclei from wild-type plants. U2B is a marker protein of the Cajal body. (**B**) Immunolocalization of STA1 and AGO4 in the nuclei from the AGO4-GFP transgenic plants. STA1 colocalizes with AGO4 in the nuclei. (**C**) Immunolocalization of STA1 relative to NRPE1 in nuclei. *NRPE1-Flag* transgenic plants were used for immunolocalization of NRPE1. The number and percentage of each type of cells are shown.

STA1 is specifically localized in nuclei (48), which is consistent with the role of STA1 in pre-mRNA splicing and RdDM. To further investigate the localization pattern of STA1 in nuclei, we subjected the nuclei extracted from the indicated plant materials for immunolocalization assay. The results show that in most nuclei (101/111, 91%), STA1 colocalizes with a nucleolus-adjacent body (Figure 8A). The body overlaps with the Cajal body signal marked by U2B (Figure 8A). Immunolocalization of AGO4-GFP indicated that AGO4 is localized in the Cajal body (Figure 8B), which is consistent with previous reports (19,60). The results suggest that STA1 colocalizes with AGO4 in the Cajal body of nuclei. In a large number of nuclei (65/107, 61%), we found that the STA1 signal in the Cajal body overlaps with NRPE1 foci when NRPE signals form condensed foci close to nucleoli (Figure 8C). Colocalization between STA1 and NRPE1 is consistent with the finding that STA1 is involved in accumulation of Pol V-dependent RNA transcripts (Figure 6B).

## DISCUSSION

The splicing factor SR45 was recently found to be required for RdDM (44). However, the functional mechanism of the splicing machinery in RdDM remains to be fully elucidated. SR45 contributes to siRNA accumulation and is likely to act during siRNA biogenesis in the RdDM pathway (44). Our study identified another splicing factor, STA1, as a new RdDM regulator. High-throughput mRNA sequencing and RT-PCR results clearly demonstrated that *sta1* has no effect on the transcripts of RdDM genes (Supplementary Table S8; Supplementary Figures S8–S10), suggesting that STA1 has a direct role in the RdDM pathway. Our results demonstrate that STA1 is required for accumulation of Pol V-dependent RNA transcripts and acts at a later step of the RdDM pathway.

Splicing factors were previously found to be required for RITS in fission yeast, but the functional mechanism is elusive (38,39). The RITS pathway in fission yeast parallels the RdDM pathway in *Arabidopsis* in that they share several evolutionarily conserved components. The finding of splicing factors in the RdDM pathway suggests that involvement of splicing factors is conserved between the RITS pathway in fission yeast and the RdDM pathway in *Arabidopsis*. A large number of hypomethylated loci caused by *sta1* as well as by *nrpd1* are present in TEs and promoter sequences (Figure 4A and B; Supplementary Table S4 and S6). Although DNA methylation is reduced by *sta1* and *nrpd1* at a small number of gene bodies, both *sta1* and *nrpd1* have no effect on the overall gene body methylation level (Figure 3A). Pre-mRNA splicing is unlikely to be directly involved in DNA methylation of corresponding genes.

STA1 is an evolutionally conserved splicing factor that is homologous to the previously characterized splicing factor PRP6, which is involved in the formation of U4/U6-U5 tri-snRNP in fission yeast and animals (48,61). Assembly of U4/U6-U5 tri-snRNP was reported to be carried out in the Cajal body (31,32). Immunolocalization analysis suggest that the *Arabidopsis* PRP6-like protein STA1 localizes in the Cajal body (Figure 8A), which is consistent with a function in pre-mRNA splicing. Moreover, AGO4, an important RdDM component, localizes in the Cajal body, indicating a critical role of the Cajal body in RdDM (19,60). Our results suggest that STA1 colocalizes with AGO4 in the Cajal body and is required for RdDM at the whole-genome level (Figures 3–6, 8B). It is possible that STA1 may facilitate assembly of the AGO4-siRNA complex in the Cajal body. Moreover, we have found that the STA1 signal in the Cajal body overlaps with NRPE1 foci in a large number of nuclei (Figure 8C), which is consistent with its involvement in production of Pol V-dependent RNA transcripts. Therefore, we propose that STA1 functions together with AGO4 and NRPE1 and acts downstream of siRNA biogenesis in the RdDM pathway.

Our small RNA deep-sequencing data suggest that the *sta1* mutation can specifically affect 24-nt siRNAs that depend on both Pol IV and Pol V, whereas it affects few of the Pol V-independent 24-nt siRNAs (Figure 5C; Supplementary Table S7). That result further supports the view that STA1 can act together with Pol V in the RdDM pathway. As expected, our semiquantitative RT-PCR demonstrated that Pol V-dependent RNA transcripts are reduced by mutation of *STA1* (Figure 6B). In fission yeast, RNA processing and elimination activities contribute to histone H3K9 methylation and transcriptional silencing on chromatin (62,63). It is worthy to note that two *Arabidopsis* RNA processing-related proteins FPA and FCA may be involved in RdDM and transcriptional silencing (64,65). The RNA processing defect in *fpa* releases the silencing of the canonical RdDM target *AtSN1* (65). Given the RNA processing ability of STA1, the involvement of STA1 in accumulation of Pol V-dependent RNA transcripts and RdDM is likely to be related to the RNA processing function of STA1. We propose that non-coding RNAs produced by Pol V are cotranscriptionally processed by STA1, and that the processed RNAs are able to act as scaffold RNAs in the RdDM pathway.

Whole-genome DNA methylation assay suggested that the effect of *sta1* is comparable with that of *nrpd1* at the whole-genome level (Figures 3 and 4). A large number of TEs are common targets for STA1 and NRPD1 (Figure 4B; Supplementary Table S4), suggesting that STA1 acts in the RdDM pathway. However, we also identified TEs whose DNA methylation is uniquely decreased by *sta1* or *nrpd1* (Figure 4B). At the unique STA1 target loci, STA1 may act on DNA methylation through an uncharacterized mechanism rather than through the RdDM pathway. Our genetic analysis indicates that the expression of the *RD29A-LUC* transgene is enhanced when the *sta1* mutation was introduced into *ros1nrpd1*, *ros1ago4* and *ros1dcl3* (Figure 7A and B), suggesting an additive effect between *sta1* and the RdDM mutations. At canonical RdDM target loci, therefore, STA1 may contribute to DNA methylation and transcriptional silencing through both RdDM-dependent and -independent mechanisms. It would be interesting to identify those factors that determine whether STA1 collaborates with the RdDM pathway or acts independently in the regulation of DNA methylation and transcriptional silencing at different chromatin regions.

## SUPPLEMENTARY DATA

Supplementary Data are available at NAR Online.

## FUNDING

National Basic Research Program of China (973 Program) [2012CB910900]; the 973 Program [2011CB812600] from the Chinese Ministry of Science and Technology and Chinese Academy of Sciences. Funding for open access charge: the 973 Program (2011CB812600) from the Chinese Ministry of Science and Technology.

*Conflict of interest statement*. None declared.

## Supplementary Material

Supplementary Data
