# Supplementary material for: The PRP6-like splicing factor STA1 is involved in RNA-directed DNA methylation by facilitating the production of Pol V-dependent scaffold RNAs
Source: Nucleic Acids Res. 2013 Jul 22;41(18):8489–502. doi: 10.1093/nar/gkt639 (PMC3794598; doi:10.1093/nar/gkt639)
Supplement: Supplementary Data [file supp_41_18_8489__index.html]

The PRP6-like splicing factor STA1 is involved in RNA-directed DNA methylation by facilitating the production of Pol V-dependent scaffold RNAs — The PRP6-like splicing factor STA1 is involved in RNA-directed DNA methylation by facilitating the production of Pol V-dependent scaffold RNAs — Supplementary Data 

# The PRP6-like splicing factor STA1 is involved in RNA-directed DNA methylation by facilitating the production of Pol V-dependent scaffold RNAs

## 

files

**Files in this Data Supplement:**

- Supplementary Data - zip file
